# Working hours associated with unintentional sleep at work among airline pilots

**DOI:** 10.1590/S1518-8787.2017051006329

**Published:** 2017-06-20

**Authors:** Elaine Cristina Marqueze, Ana Carolina B Nicola, Dag Hammarskjoeld M D Diniz, Frida Marina Fischer

**Affiliations:** I Departamento de Epidemiologia. Programa de Pós-Graduação em Saúde Coletiva. Universidade Católica de Santos. Santos, SP, Brasil; II Associação Brasileira de Pilotos da Aviação Civil. São Paulo, SP, Brasil; III Departamento de Saúde Ambiental. Faculdade de Saúde Pública. Universidade de São Paulo. São Paulo, SP, Brasil

**Keywords:** Aviation, Human Resources, Sleep Deprivation, Epidemiology, Working Conditions, Shift Work, Occupational Health

## Abstract

**OBJECTIVE:**

Tto identify factors associated with unintentional sleep at work of airline pilots.

**METHODS:**

This is a cross-sectional epidemiological study conducted with 1,235 Brazilian airline pilots, who work national or international flights. Data collection has been performed online. We carried out a bivariate and multiple logistic regression analysis, having as dependent variable unintentional sleep at work. The independent variables were related to biodemographic data, characteristics of the work, lifestyle, and aspects of sleep.

**RESULTS:**

The prevalence of unintentional sleep while flying the airplane was 57.8%. The factors associated with unintentional sleep at work were: flying for more than 65 hours a month, frequent technical delays, greater need for recovery after work, work ability below optimal, insufficient sleep, and excessive sleepiness.

**CONCLUSIONS:**

The occurrence of unintentional sleep at work of airline pilots is associated with factors related to the organization of the work and health.

## RESUMO

**OBJETIVO:**

Identificar fatores associados aos cochilos não intencionais durante as jornadas de trabalho de pilotos da aviação regular.

**MÉTODOS:**

Estudo epidemiológico transversal conduzido com 1.235 pilotos brasileiros de avião do transporte aéreo regular, que realizavam voos nacionais ou internacionais, sendo a coleta de dados realizada *on-line*. Foi realizada análise de regressão logística bivariada e múltipla, tendo como variável dependente o cochilo não intencional durante o horário de trabalho. As variáveis independentes foram relacionadas a dados biodemográficos, características do trabalho, estilo de vida e aspectos do sono.

**RESULTADOS:**

A prevalência do cochilo não intencional enquanto pilotava o avião foi de 57,8%. Os fatores associados ao cochilo não intencional foram: voar por mais de 65 horas por mês, atrasos técnicos frequentes, maior necessidade de recuperação após o trabalho, capacidade para o trabalho inferior à ótima, sono insuficiente e sonolência excessiva.

**CONCLUSÕES:**

A ocorrência do cochilo não intencional durante a jornada de trabalho de pilotos da aviação regular está associada a fatores relacionados à organização do trabalho e à saúde.

## INTRODUCTION

The Brazilian civil aviation, according to the 2013 Air Transport Directory of the Brazilian Civil Aviation Agency (ANAC)^a^, has underwent significant changes in operational management in recent years, directly impacting flight operations. The fleet of Brazilian airlines reached 563 airplanes by the end of 2013, representing an increase of 8.7% from 2012. The number of passengers carried has reached the highest number in history: more than 109 million, being 90 million in domestic flights and 19.2 million in international flights^a^. However, the number of employees in the companies followed the opposite direction, with a reduction in the number of pilots^a^. The average number of employees per airplane went from 118 to 106.3, indicating that the decrease is a trend of the aviation industry. This reduction reached both the support staff on the ground and crew (flight crew and cabin crew). Currently, pilots (captains and first officer) represent approximately 10% of the staff of airline companies^a^.

Given this scenario, the fatigue and irregular and long working hours (usually above eight hours per day) of pilots raises concern about the safety of air operations^4^ ^,^ ^17^. The flights that start at dawn, those that end late at night, night flights, and the crossing of time zones change the sleep-wake cycle, the alert levels, and the decision-making of pilots during flights^12^. These factors can cause excessive sleepiness throughout the workday, which increases the propensity for unintentional sleep at work and risk of incidents or accidents at work^2^ ^,^ ^9^. Ingre et al.^12^ have observed that unintentional sleep at work is a reflection of the work conditions and organization of pilots. The authors^12^ have also shown that unintentional sleep at work may compromise the safety of flights.

In fact, the work activities of pilots are complex and require multiple skills, both technical and relational ones. Among them we can mention the ability to concentrate, work under pressure, adapt to operational changes, work as a team, anticipate the consequences of a set of signals, and interpret these signals for fast decision making^13^. With excessive sleepiness, some of these skills can be compromised, and with them, the safety of flights.

In this context, the objective of this study has been to identify factors associated with unintentional sleep at work of airline pilots.

## METHODS

This is a cross-sectional epidemiological study conducted with airline pilots, affiliated to the Brazilian Association of Civil Aviation Pilots (ABRAPAC) and who work on the five major Brazilian airlines. The target population (all pilots associated with ABRAPAC) amounted to 2,530 pilots at the time of data collection. According to the data of ANAC^a^, this number represented approximately half of the Brazilian airline pilots. We sent an individualized invitation using e-mail to the 2,530 pilots for participation in the study. The online data collection was carried out from December 2013 to March 2014, and during this period we resent the invitations to participate systematically every 15 days. Approximately 1,234 pilots working in national or international flights participated in this investigation, amounting to 48.8% of the original sample. This was a convenience sample ,since only the pilots associated with ABRAPAC were invited to participate.

After reading the informed consent, the pilots should register online that they agreed to participate in the study; only after their agreement they could respond to the questionnaire. Because it was an online questionnaire, the pilots could respond partially to the questionnaire and finish it whenever it was convenient. This was a factor which contributed to increase their participation. This study was approved by the Research Ethics Committee of Instituto Federal de Educação, Ciência e Tecnologia de São Paulo (Protocol 625,158).

The independent variables were: biodemographic data (gender, marital status, children under 12 years of age, number of persons who contribute to the family income, education), characteristics of the work (time working as pilot, time working in the current company, position place of residence, type of crew, frequency of flight delays, type of day off, work shift, and monthly hours of flight, home/airport standby, and on call^b^, commuting time (between residence and contractual basis, commuting time between hotel and airport), duration of working hours, number of consecutive days and nights of work, number of legs per day, time working on the night shift, starting and ending times of working hours, number of days off, factors that cause fatigue at work, quality of the place available for sleep inside the airplane and hotels provided by the company), lifestyle (smoking and alcohol consumption evaluated by AUDIT – Alcohol Use Disorders Identification Test^20^) and aspects related to sleep (described below).

The dependent variable “unintentional sleep at work” during work was evaluated by a single question (“have you ever unintentionally slept while flying the airplane?”), with the option to answer yes or no. This question was been adapted from the Karolinska sleep questionnaire (Unintended periods of sleep – nodding off *–* at work)^1^, in which nap is regarded as any short period of sleep that is less than half the length of the main sleep, and it may be a few seconds or minutes. The questions of how they sleep and if they sleep enough, the index of problems to wake up, and the sleep quality index were also adapted from the Karolinska sleep questionnaire^1^. We used the Berlin questionnaire to estimate the chance of developing Obstructive sleep apnea syndrome^15^. To evaluate excessive sleepiness, we used the Epworth sleepiness scale^15^.

To evaluate the need for recovery after work, we used the scale proposed by Veldhoven and Broersen^24^, which have a score from zero to 100, proportional to the need for recovery. The physical and mental work ability was evaluated by the Work Ability Index (WAI) developed by Tuomi et al.^23^ The WAI provides a score that ranges from 7 to 49 points, and it has seven dimensions (current work ability, work ability in relation to the requirements of the work, current number of diseases diagnosed by a physician, estimated loss of work ability because of illnesses, absences from work because of illness in the last 12 months, self-prognosis on work ability for the next two years)^23^.

Being unintended sleep at work the outcome variable, the sampling power of this research was 88%, considering a sampling error of 5% (G*Power, Version 3.1.4). Normality of the variables was tested suing the Shapiro-Wilk test. We described the simple and relative frequencies of the studied variables and performed analyses of bivariate and multiple logistic regression, with the dependent variable being having unintentionally slept at work. We used the Hosmer-Lemeshow test to test the goodness of fit of the model. In all tests, we considered as significant if p < 0.05. For the statistical analyses, we used the program Stata 12.0 (Stata Corp., Texas, USA).

## RESULTS

Most participants (97.1%) were males, had a partner (84.3%), and did not have children younger than 12 years (61.3%). The average age of the pilots was 39.1 years (SD = 9.8 years). The number of persons who contributed to the family income was, on average, 1.6 persons (SD = 0.7).

Most pilots (82.4%) were attending or had already completed college education. Working time as a pilot was on average 15.2 years (SD = 10.1 years) and the average working time on current airline was 5.8 years (SD = 4.8 years). Most of the respondents worked as captain (57.9%), and the others were first officer (42.1%); 53.7% did not reside in the same location of the contractual basis. Almost all participants (91.2%) reported flying almost always with simple crew^c^ (91.2%).

A high percentage of pilots reported that delays occurred frequently or always because of operational, maintenance, and dispatch issues (40.7%). The type of time off varied between the pilots, but for 27.6% they were only one day off. The work shift of almost all pilots (94.1%) was irregular and involved the night shift – from 10 p.m. to 5 a.m.

The average number of monthly flight hours was approximately 65 hours. Commuting time between residence and contractual basis was almost three times higher among those who lived outside the contractual basis compared to those who lived in the same location as the basis. We highlight that most resided outside their contractual basis. Working hours^d^ were longer during the day shift, followed by the afternoon and night shifts (Table 1).

**Table 1:** Means and standard deviations of monthly working hours, commuting time, work days, and days off among Brazilian airline pilots.

Variable	n	Average	SD
Flight hours (hours/month)	1,231	64.8	10.8
Hours of standby (hours/month)	1,157	9.2	6.6
Hours of on call (hours/month)	1,164	22.3	15.3
Commuting time from the residence to the contractual basis for the pilots who do not reside in the same location of the basis (min)	645	161.4	121.5
Commuting time from the residence to the contractual basis for the pilots who reside in the same location of the basis (min)	566	58.2	43.4
Commuting time hotel x airport (min)	1,230	41.1	25.0
Working hours – day shift (hours)	1,198	8.8	1.4
Working hours – afternoon shift (hours)	1,127	8.0	1.7
Working hours – night shift (hours)	1,160	7.8	2.4
Number of days off per month (days/month)	1,234	9.2	1.4
Maximum number of consecutive days of work	1,226	6.1	1.2
Maximum number of consecutive nights of work	1,187	4.0	2.4
Maximum number of working hours in the same day	1,193	4.9	1.1
Time working in the night shift (years)	558	9.6	7.7

Regarding working hours in the day, afternoon, and night shifts, day shift had a higher proportion of pilots who started their work before 6 a.m. The end of work in the afternoon shift was usually after 10 p.m. In the night shift, in turn, the start time had a higher proportion before 10 p.m. (Table 2).

**Table 2:** Work schedule among Brazilian airline pilots.

Hours	n	%
Start of work in the day shift		
From 12 a.m. to 4h59 a.m.	220	18.4
From 5 a.m. to 5h59 a.m.	584	48.7
From 6 a.m. to 6h59 a.m.	254	21.2
From 7 a.m. to 11 a.m.	140	11.7
End of work in the afternoon shift		
From 4 p.m. to 9h59 p.m.	403	35.8
From 10 p.m. to 11h59 p.m.	562	49.9
From 12 a.m. to 6 a.m.	162	14.3
Start of work in the night shift		
From 2 p.m. to 9h59 p.m.	687	59.3
From 10 p.m. to 11h59 p.m.	393	33.9
From 12 a.m. to 1h59 a.m.	44	3.8
From 2 a.m. to 4h30 a.m.	35	3.0
End of work in the night shift		
Before 5h a.m.	598	51.6
From 5h01 a.m. to 8 a.m.	423	36.5
From 8h01 a.m. to 12 p.m.	127	11.0
From 12h01 p.m. to 4 p.m.	10	0.9

Regarding the working conditions that could be associated with increased fatigue, the main factors reported by the pilots were: long working hours, flight hours, little rest time between working hours, and night work (Figure).

**Figure:**
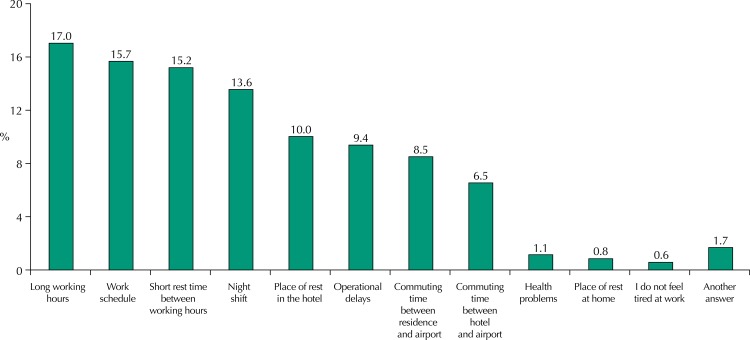
Perception of factors that cause fatigue at work among Brazilian airline pilots.

The pilots evaluated their in-flight cabin for rest in relation to light, noise, thermal comfort, and physical comfort. The average was 2.82, 2.14, 4.07, and 1.85 points, respectively. The quality of the hotels provided by airlines for rest was also evaluated using the same parameters and the averages were higher when compared to the places of the airplane (5.84, 5.25, 6.66, and 6.24 points, respectively).

The percentage of smokers was low (7%). The proportion of pilots who reported drinking alcohol was 75%. Among those who drink, low-risk use was reported by 75.2%, moderate use by 23.6%, and harmful use or possible dependence was reported by 1.2%.

Among the 1,234 pilots, 578 diseases with medical diagnosis were reported, being the most prevalent diseases injuries, musculoskeletal disorders, and digestive and respiratory diseases (35.8%, 28.2%, 24.6%, and 21.6%, respectively).

The average time reported for sleep latency (time between lying down and the beginning of sleep) after working on the day shift was 40.7 minutes, and 38.3 minutes after the night shift. The duration of sleep after work on day and night shifts was 6.9 hours (SD = 1.2 h) and 6.7 hours (SD = 1.9 h), respectively. On days off, duration of sleep was higher than in working days (8.8 hours). When asked whether they sleep enough, 31.2% reported that they sleep little. On the subjective quality of sleep, 10.6% of the pilots classified it as bad. The average percentage of need for recovery after work on a scale from zero (less need) to 100% (greater need) was 61.2% (SD = 26.4%).

Most pilots reported that they have unintentionally slept while they were in command of the airplane (57.8%). Table 3 presents the factors associated with unintentional sleep. In the adjusted multiple model, those who flew for more than 65 hours a month showed increased 78% chance of unintentionally sleeping while flying the airplane when compared to pilots who had average monthly flight time of 65 hours or less. Frequent flight delays and greater need for recovery increased the chance of unintentional sleep at work in 45% and 49% compared to those who reported fewer operational delays and reduced need for recovery after work, respectively. Work ability being less than optimal was another factor associated: the worse the work ability, the greater the chance of sleeping, reaching almost five times more in the category “low work ability”. Sleep perceived as insufficient and excessive sleepiness increased by 44% and by more than two times, respectively, the chances of unintentional sleep while piloting, compared to those who perceived having enough sleep and presented low sleepiness.

**Table 3:** Bivariate and multiple logistic regression for unintentional sleep at work.

Variable	Category	%	OR	95%CI	adj OR^a,b^	95%CI
Factors related to work						

Responsibility for family income	Shared with someone	52.4	1			
	Alone	47.6	1.07	0.84–1.35		
Work shift	Day	5.6	1			
	In legs that involve nights	94.4	1.53	0.94–2.49		
Flight routes	National	90.8	1			
	International	9.2	**1.78**	**1.17–2.71**		
Time working in the night shift	Does not work all night	54.8	1			
	1 to 5 years	17.8	1.12	0.83–1.52		
	6 to 10 years	13.3	**1.49**	**1.05–2.12**		
	11 to 15 years	5.5	**2.80**	**1.57–5.01**		
	16 years or more	8.6	**1.75**	**1.14–2.69**		
Monthly flight hours	Up to 65 hours	47.0	1		1	
	66 hours or more	53.0	**2.19**	**1.74–2.76**	**1.78**	**1.38–2.29**
Maximum number of consecutive days of work	Up to 6 days	81.6	1			
	7 days or more	18.4	**2.33**	**1.69–3.21**		
Maximum number of consecutive nights of work	One or two nights	17.2	1			
	Three or four nights	56.4	1.23	0.90–1.69		
	Five or more nights	26.4	**1.60**	**1.12–2.29**		
Average days off per month	10 days or more	41.7	1			
	Up to 9 days	58.3	**1.71**	**1.36–2.15**		
Frequency of operational flight delays	Never, rarely, or sometimes	59.4	1		1	
	Frequently or always	40.6	**2.06**	**1.62–2.61**	**1.45**	**1.11–1.89**
Commuting time between hotel and airport	Up to 42 minutes	68.8	1			
	42 minutes or more	31.2	**1.55**	**1.21–1.99**		

Aspects of sleep						

How do you sleep?	Very well or well	51.9	1			
	Neither well, nor bad	37.5	**1.63**	**1.28–2.09**		
	Fairly or very bad	10.6	**2.87**	**1.88–4.38**		
Do you sleep enough?	Yes	68.9	1		1	
	No	31.1	**2.20**	**1.70–2.84**	**1.44**	**1.07–1.94**
Quality of the hotel to sleep (noise, lighting, and thermal and physical comfort)	Above average	51.8	1			
	Below average	48.2	**1.47**	**1.17–1.84**		
Quality of the sleeping cabin in the airplane (noise, lighting, and thermal and physical comfort)	Above average	47.7	1			
	Below average	52.3	**1.34**	**1.07–1.69**		
Index of problems to wake up	No	82.3	1			
	Yes	17.7	**2.28**	**1.65–3.16**		
Sleep quality index	Good	92.4	1			
	Bad	7.6	**2.49**	**1.52–4.08**		
Obstructive sleep apnea syndrome	Low chance of developing	79.7	1			
	High chance of developing	20.3	**2.03**	**1.50–2.74**		
*Epworth score*	Low sleepiness	58.1	1		1	
	Excessive sleepiness	41.9	**2.80**	**2.20–3.57**	**2.09**	**1.60–2.72**

Health						

Scale of the need for recovery after work	Less need	31.8	1		1	
	Moderate need	34.7	**1.72**	**1.30–2.26**	1.06	0.77–1.47
	Greater need	33.5	**3.30**	**2.46–4.42**	**1.49**	**1.04–2.16**
Work ability index	Great	33.7	1		1	
	Good	45.7	**2.20**	**1.70–2.85**	**1.52**	**1.13–2.04**
	Moderate	18.2	**3.94**	**2.76–5.63**	**2.15**	**1.43–3.23**
	Low	2.4	**8.95**	**3.07–26.11**	**4.80**	**1.57–14.67**

^a^ Adjusted for age, education, gender, marital status, and position

^b^ Hosmer-Lemeshow = 0.94

Values with statistical significance are presented in bold.

## DISCUSSION

The occurrence of unintentional sleep at work was the main outcome of interest of this study. We observed a significant association with factors related to the organization of work (number of monthly flight hours, operational delays, need for recovery after work) and health (work ability, insufficient sleep, and excessive sleepiness).

The percentage of pilots who reported having unintentionally slept during flight was high and twice as higher than that found by Gregory et al.^10^ in an American population study with pilots of medical operations. Our finding is relevant, as unintentional sleep at work may compromise the safety of flights and it is a reflection of the conditions and organization of work of Brazilian airline pilots^12^. According to Cabon et al.^3^, pilots who fly long distances are particularly susceptible to lapses during vigil, as well as during periods of low workload. Such lapses can occur simultaneously with both pilots responsible for the flight.

As perceived by the participants of this study, most factors that cause fatigue at work are: the hours of work, long daily working hours, night work, and reduced resting time between working days. These factors were also observed in other studies with crews^4^ ^,^ ^17^ ^,^ ^18^. Although irregular working hours and night shift have not been associated with unintentional sleep at work in this study, they are contributing factors for the perception of fatigue and excessive sleepiness, according to what has been reported in previous studies^4^ ^,^ ^17^ ^,^ ^18^.

As flight hours are irregular, one factor to be highlighted is the starting and ending times of daily work. When working on day shifts, pilots may start their work at dawn. In addition, if we also consider commuting time (home × workplace, hotel × workplace), the duration of the sleep is impaired, especially when waking up in time of greater sleepiness (around 3 a.m. or 4 a.m.)^2^.

Approximately half of the pilots participating in the study did not live in the same city as their contractual basis, which increases commuting time up to three times to the workplace and return to the residence on days off, compared to those who live in the same town of their contractual basis. Commuting time is not taking into account when calculating the working hours. However, this variable can be associated with the perception of insufficient sleep and worsen fatigue, since it decreases the time to rest during break(s), or between successive work days^4^ ^,^ ^17^.

Another factor that deserves attention is the number of consecutive nights at work, which on average was four nights. According to the studies on shift work, the risk of incidents or accidents when working four consecutive nights is approximately 36% higher compared to only one night of night work^7^. In this context, Rodionov^19^ have suggested allowing only two night shifts in a row at most, since three successive night shifts increase the risk of chronic fatigue.

According to current regulations (Law 7,183 of April 5, 1984), flight hours reported by the participants of this study did not exceed those provided by law (85 h/month). However, the monthly average flight hours above 65 hours were associated with unintentional sleep while piloting. We highlight that we did not collect information on the maximum duty time, which include times for presentation, engine shutdown, and duration of operating delays. Therefore, the results observed in this study on the actual flight hours are less than maximum duty time. One aspect that hinders the use of flight hours is the operational flight delays, which occur frequently. It is worth noting that this variable was associated with unintentional sleep while flying the airplane.

Caldwell^4^ and Powell et al.^17^ draw attention to the multiple negative effects associated with insufficient sleep and fatigue. In this study, a high proportion of the pilots felt that they need to sleep more. In fact, sleep on days off was longer than on working days, indicating a daily sleep debt of approximately 2 hours.

Unintentional sleep at work is added to other factors that are rarely mentioned in studies already carried out, such as the quality of resting places, hotels, and cabins of airplanes^e^. In our study, on a scale from zero to 10, the participants rated as very bad the rest cabins inside airplanes, and only as regular the quality of hotel rooms.

The need for recovery after work reported by the pilots was higher in comparison to other professional categories, being associated with unintentional sleep at work^22^ ^,^ ^21^. These data indicate that the work as a pilot, because of its characteristics, such as irregular working hours and the complexity of the work^4^ ^,^ ^5^ ^,^ ^17^, requires a longer time for recover than those stipulated in the Brazilian law (at least 12 hours of rest between two consecutive working days). Australian researchers^18^ suggest at least two consecutive nights of rest for recovery after night work, being extended to four nights after international flights. The regulation of the profession (Law 7,183 of April 5, 1984) establishes a period of rest according to flight time, type of crew, previous flight hours, number of time zones crossed, and work shift. However, considering the Brazilian territorial extension, the current legislation does not provide for sufficient time for recovery after work, especially for night work or work which starts very early in the morning.

Predominantly, pilots had one day off (which lasts 12 hours after a workday plus 24 hours off). Even considering that the current legislation on the working hours is being respected, the irregularity of flight hours associated with simple time off is aggravating in the issues related to sleep and, consequently, health^19^.

The work ability index (WAI) has been used as a variable to predict the health condition of workers^23^. In our study, we observed that the worse the WAI, the greater the chance of unintentional sleep at work. These data are of concern, as this is a population of young adults considered as healthy, as a rigorous and annual medical evaluation is required to stay on active duty, and when pilots reach 40 years of age, this evaluation occurs every six months.

The pilots reported a significant proportion of diseases diagnosed by physicians, being the most prevalent injuries and musculoskeletal disorders. However, the prevalence of these injuries and musculoskeletal disorders are below those mentioned in other studies^8^ ^,^ ^11^. Research carried out 22 years ago in a Brazilian airline already indicated the concern of pilots with their health and precarious work conditions^6^. The pilots identified the need to review the criteria used in the preparation of work shifts and concepts established in the professional regulation, such as rest periods between consecutive working days and days off^6^. The regulation of the profession at the time of completion of that study is the same in force (Law 7,183 of April 5, 1984), that is, the legislation has been not reviewed or amended for more than 30 years despite the growth of the civil aviation.

The sample of the study may be under-represented, because only pilots associated with ABRAPAC participated in it. We do not have information on the sociodemographic characteristics of those who did not participate in the study, which may also have led to a bias in the results. The cross-sectional design of this study does not allow us to make causal inferences between the variables analyzed. Despite the limitations mentioned, an expressive number of pilots participated, which allows us to draw a picture of the working conditions and possible effects on the health and sleep of Brazilian airlines pilots.

Factors related to work, such as flight hours, inappropriate use of working hours because of operational delays, and poor working conditions, need to be taken into account to prevent unintentional sleep at work. In addition to these aspects, it is necessary to provide a sufficient time for recovery between working hours, as the activities carried out by pilots are complex, requiring significant demands of work with a cognitive nature.

The results of this study highlight the need to implement preventive actions related to the organization of work, aiming at improving working conditions and health, especially in aspects related to the sleep of pilots. As one of the preventive practices to relieve excessive sleepiness during the work, scheduled naps before and during flights are recommended, especially on night or long-haul flights^16^ ^,^ ^25^. Although sleep is allowed during the flight when there is a composed or flight crew, most flights of the respondents of this study had simple crew. Brazilian airlines have waiting rooms for their flight crews, but not all have places to sleep. It is necessary to improve the facilities for rest, whether they are on the plane in itself, in hotels, or at airports.

Work schedules should follow the recommendations proposed by Rodionov^19^ and Roach et al.^18^ to prevent unintentional sleep at work. It is worth mentioning that the beginning of the work schedule during the early hours and the late ending are also harmful to health. Thus, we recommend the reduction of the number of working hours that begin very early in the morning, as well as that end very late at night. Airlines can benefit from integrated actions in the planning of work schedules, forming teams with members of the industry who operate in the construction of working plans including health, safety, and environment professionals, as well as representatives of the airline pilots.

## Footnotes

a Agência Nacional de Aviação Civil. Anuário do Transporte Aéreo 2013. Brasília (DF): ANAC; 2013 [cited 2016 Dec 7]. Available from: http://bibspi.planejamento.gov.br/bitstream/handle/iditem/628/Anu%C3%A1rio%20do%20Transporte%20A%C3%A9reo%20de%202013.pdf?sequence=1

b Brasil. Lei 7.183, de 5 de abril de 1984. Regula o exercício da profissão de aeronauta e dá outras providências. Brasília (DF); 1984. Capítulo II; Seção III: Do Sobreaviso e Reserva. Available from: https://www.planalto.gov.br/ccivil_03/Leis/L7183.htm. Article25 - “on call” is the period of time that does not exceed 12 hours, in which crews remain in the place of their choice, available to the employer, and they must report to the airport or another determined location, up to 90 minutes after receiving communication to initiate a new assignment. Paragraph 1- The number of on call periods that the crew may have should not exceed two per week or eight per month. Article26 - “Standby” is the period of time in which the crew remains, by determination of the employer, available in the workplace . Paragraph 1- The period of standby of crews of airline companies must not exceed six hours. Paragraph 3 – The employer must ensure appropriate accommodations for the crew to rest if a planned standby lasts longer than three hours.

c Simple crew basically consists of a minimum crew plus, if applicable, the crew needed to carry out the flight (Brazil. Lei 7.183, de 5 de abril de 1984. Regula o exercício da profissão de aeronauta e dá outras providências. Brasília (DF); 1984. Capítulo 1; Seção II: Das Tripulações. Available from: https://www.planalto.gov.br/ccivil_03/Leis/L7183.htm)

d According to Law 7,183 of April 5, 1984, working hours are counted from the time of presentation at the workplace (contractual basis), or at the place determined by the employer (outside the contractual basis). Working hours end 30 minutes after the final stop of the motors (Brazil. Lei 7.183, de 5 de abril de 1984. Regula o exercício da profissão de aeronauta e dá outras providências. Brasília (DF); 1984. Capítulo II; Seção II: Da Jornada de Trabalho. Available from: https://www.planalto.gov.br/ccivil_03/Leis/L7183.htm)

e Rosekind MR, Gregory KB, Co EL, Miller DL, Dinges DF. Crew factors in flight operations XII: a survey of sleep quantity and quality in on-board crew rest facilities. Moffett Field: NASA; 2000 [cited 2016 Nov 29]. Report No. NASA/TM-2000-20961. Available from: https://ntrs.nasa.gov/archive/nasa/casi.ntrs.nasa.gov/20010038243.pdf
